# On the passivation of iron particles at the nanoscale†

**DOI:** 10.1039/c9na00161a

**Published:** 2019-04-23

**Authors:** Maximilian Lasserus, Daniel Knez, Martin Schnedlitz, Andreas W. Hauser, Ferdinand Hofer, Wolfgang E. Ernst

**Affiliations:** Institute of Experimental Physics, Graz University of Technology Petersgasse 16 A-8010 Graz Austria andreas.w.hauser@gmail.com wolfgang.ernst@tugraz.at +43-316-873-108140 +43-316-873-8157 +43-316-873-8140; Institute for Electron Microscopy and Nanoanalysis & Graz Centre for Electron Microscopy, Graz University of Technology Steyrergasse 17 A-8010 Graz Austria

## Abstract

The oxidation of Fe@Au core@shell clusters with sizes below 5 nm is studied *via* high resolution scanning transmission electron microscopy. The bimetallic nanoparticles are grown in superfluid helium droplets under fully inert conditions, avoiding any effect of solvents or template structures, and deposited on amorphous carbon. Oxidation resistivity is tested by exposure to oxygen at ambient conditions. The passivating effect of Au-shells is studied in detail and a critical Au shell thickness is determined which keeps the Fe core completely unharmed. Additionally, we present the first synthesis of Fe@Au@Fe-oxide onion-type structures.

## Abstract 2

The oxidation of Fe@Au core@shell clusters with sizes below 5 nm is studied *via* high resolution scanning transmission electron microscopy.

## Graphical abstract

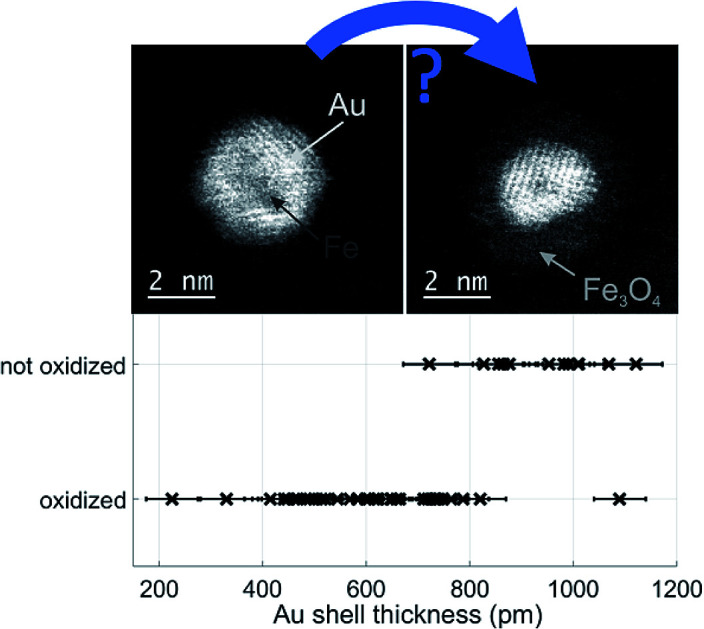

## 1 Introduction

Core@shell nanoparticles represent a class of materials with unique physical properties and various fine-tuning possibilities *via* an adjustment with respect to size, morphology and composition. Due to this extreme flexibility, a wide range of potential applications has been suggested for these materials.^1–5^ In medical sciences, nanostructures with magnetic properties have become particularly interesting for potential application in diagnosis, drug delivery, cell separation, thrombolysis and cancer treatment.^6–11^ Preferred are particles below 20 nm diameter due to enhanced tissular diffusion in this size regime.^12^

Iron-based nanostructures have been studied thoroughly for applications as magnetic agents.^13,14^ Qiang *et al.* could prove that iron clusters with a non-oxidized Fe core, passivated by a shell of iron oxide, have a much higher magnetic moment than fully oxidized clusters.^15^ The latter type could be synthesized by exposure to oxygen during particle synthesis. Fe clusters which are exposed to oxygen after synthesis show characteristic cavities,^16^ a result of Kirkendall dynamics, an effect observed for several metals:^17^ metal atoms inside the cluster are dragged towards the surface where oxidation takes place.

In this context, a passivation with a layer of gold seems reasonable in order to retain a highly magnetic core. Besides, the combination of a magnetic material and a heavy element providing a high X-ray contrast offers the opportunity to design dual agents suitable for both X-ray and MRI diagnostics. Among other passivation choices, Au stands out because of its high bio-compatibility,^18^ optical properties,^19,20^ inertness and the ability to adsorb molecules to its surface.^21^ Additionally, Au nanoparticles have been suggested as dual agents suitable for X-ray and optical detection,^22^ due to their plasmonic properties.^23,24^ With regards to medical applicability, combinations of Fe and Au hold a high potential for future use,^25^ and several attempts have been made to synthesize various combinations of Fe and Au structures, including dumbbells,^26^ core@shell^27^ and yolk–shell clusters.^28^ For the purpose of passivation, core@shell structures are obviously most feasible. Recently, we could show that inter-metallic diffusion in core@shell nanoparticles is not only dependent on the temperature but also on the surrounding gas: the mere presence of molecular oxygen can enhance the mobility of the reactive core metal.^29^

In this article, we extend our investigation towards the passivation of the oxygen-sensitive Fe core by an additional coating with several layers of Au atoms. Helium-droplet based synthesis is particularly convenient for unbiased studies of reactivities as it provides a solvent-free and fully inert particle growth. Its underlying principle is the controlled pickup of metal atoms from vapor *via* a beam of superfluid helium droplets. The latter have been extensively utilized in the past as superfluid, inert and extremely cold environments for the spectroscopy of atoms, molecules and small clusters.^30–32^ In our current setup, we perform a sequential doping with Fe followed by Au in order to synthesize mixed-metallic Fe@Au core–shell nanoparticles at fully inert conditions inside the helium droplets, fully protected from any gas phase reactions and without templates or solvents affecting their growth. The synthesis is followed by a deposition under soft landing conditions^33^ onto amorphous carbon TEM grids, allowing us a controlled creation^34,35^ and deposition of Fe@Au clusters with a mean diameter of ≤5 nm. The clusters are first exposed to air for 120 minutes and then examined with high resolution Scanning Transmission Electron Microscopy (STEM)to evaluate their resistivity to oxidation. Since oxidation effects for nanoparticles below 10 nm at ambient conditions take place at timescales of a few minutes,^36^ exposition times in the hour range are expected to enforce a full oxidation of any unprotected reactive metal. We determine a critical minimum thickness of the gold shell necessary to protect the iron core and give an explanation of our findings based on density functional theory.

## 2 Experimental setup

For a comprehensive overview of the experimental setup we refer to ref. 37. A schematic can be found in the ESI.† He gas with a purity of 99.9999% and a pressure of 20 bar is expanded through a 5 μm nozzle which is cooled to cryogenic temperatures, resulting in the formation of a beam of helium nanodroplets. For the chosen nozzle temperature of 8 K the droplets have a mean diameter of ≈50 nm.^30^ After expansion and further collimation with a 400 μm skimmer the resulting He droplet beam enters the pickup chamber (≈10^−7^ mbar). Here, the helium droplets collect Fe and Au atoms sequentially while crossing two separate resistively-heated pickup cells. The probability for a particle pickup in each of these cells is controlled by the cell temperature, which allows us to adjust the total amount of atoms collected by the helium droplet beam as well as their mixing ratio. Atoms captured by the helium droplet beam start to agglomerate to clusters within the droplet during the flight through the vacuum chambers. The release of binding and kinetic energy of the captured atoms leads to the evaporation of helium. This attenuation of the beam is monitored by a residual gas analyzer (Balzer QMA 200/QME 200). At the end of the pickup chamber, the beam of helium droplets is again collimated by a 2 mm aperture before entering the measurement chamber with a base pressure of ≈5 × 10^−9^ mbar. In this chamber, the helium droplet beam hits the TEM grid in a soft-landing process damped by the evaporation of He during impact.^38–40^ Additional information on the surface coverage is obtained by mass deposition measurements with a microbalance.

### 2.1 Nanoparticle synthesis

The ultracold He environment allows for a controlled synthesis of core@shell structures *via* sequential doping.^41^ Depending on the original He droplet size before the pickup of metal atoms and the vapour pressure in the pickup cells, it is possible to create either spherical or elongated nanostructures. In the current manuscript, the parameters of 8 K nozzle temperature and 20 bar pressure are used, which corresponds to an average size of *N̄* ≈ 10^7^ helium atoms per helium droplet.^30^ For this choice of He containers, metal clusters of approximately spherical shape are produced with diameters in the nanometer range.^42^ The core@shell structure arises from the sequential doping of the droplet and the coagulation of the first element before enclosure by the second element. The size of the clusters follows a log-normal distribution.^43^ We choose three different doping ratios of 25 at%, 50 at% and 70 at% core (Fe), corresponding to 75 at%, 50 at% and 30 at% of shell material (Au). On average, clusters with a diameter of 3.5 nm are produced, consisting of approximately 2500 atoms per cluster. Further information on the average thickness of the Au layer for the various doping ratios can be found in the ESI,† which also gives an overview of the overall distribution of particles sizes obtained in the experiment.

After deposition on an amorphous carbon TEM grid (Ted Pella 01824G), the particles remained under UHV conditions for 48 h before being removed from the chamber and left under ambient conditions for 120 minutes in order to examine the process of oxidation.

### 2.2 Data acquisition

The particles are studied *via* a scanning transmission electron microscope (STEM, FEI Titan^3^ G2 60-300, operating at 300 kV) using a high-angular annular dark-field (HAADF) imaging detector (Fischione Model 3000). A Gatan quantum energy filter attached to the microscope is employed for electron energy loss spectroscopy (EELS). Complementary, a four-quadrant energy-dispersive X-ray spectroscopy (EDX) detector (FEI Super-X) is used. Note that the intensity of the elements in the HAADF images is proportional to approximately *Z*^2^ (with *Z* denoting the atomic number), leading to highest intensities for the Au atoms.^44^

## 3 Results

**Fig. 1:**
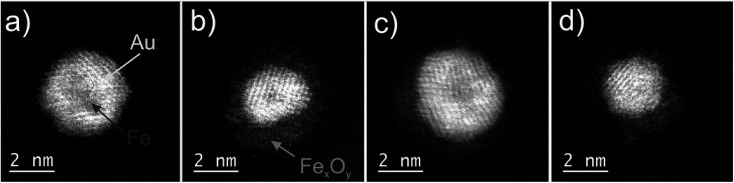
STEM HAADF images of various types of core@shell clusters. From left to right: (a) Fe@Au core@shell structure (featuring a complete shell, *i.e.* a fully passivated iron core), (b) Fe@Au@Fe-oxide core@shell@shell, (c) Fe@Au with an incomplete additional shell of iron oxide, (d) Au cluster adjacent to a Fe_3_O_4_ cluster. The first three configurations are all related to a Fe@Au core@shell structure, see text for details. The Janus-type structure in the fourth picture is an example of incomplete coating due to a lack of Au atoms.

Following the synthesis described above, a variety of more or less oxidized FeAu cluster structures is obtained after exposure to air. Four different species are depicted in Fig. 1. Their chemical structure can be clearly identified based on the contrast in the HAADF images, which is lower for metal oxides than for pure metals: picture (a) shows a pristine Fe@Au cluster, while image (b) depicts a partially oxidized particle which is best described as an onion-like structure denoted as Fe@Au@Fe-oxide. It contains a small intact iron core, covered by a first shell with enhanced contrast, identified as Au, and a second shell of lowest contrast, corresponding to iron oxide. It can be safely assumed that the Fe must stem from the core due to the order of element pickup in the process of cluster synthesis described above. Therefore, the onion structure is a clear indication of a partial leaking of iron from the core towards the surface, despite the Au coating, which renders the encapsulation as either ineffective or incomplete in these cases. For some clusters, also the oxide shells themselves appear as not completely closed, as can be seen in picture (c) of Fig. 1. Other particles, *e.g.* the cluster in picture (d), are fully oxidized and do not show a layered structure anymore. Instead, they resemble a Janus-type configuration, a structural feature which has attracted much interest in the last decade due to its potential for applications as catalysts or biosensors.^45,46^ Their occurrence is explained by the log-norm distribution of cluster sizes, which allows for situations where the amount of Au collected in the pickup process is insufficient to cover the Fe core entirely. A larger percentage of Janus particles can be enforced by a higher ratio of Fe to Au during pickup.

An interesting finding concerns the oxidation process itself and its consequence for the nanoparticle structure. Typically, when the Fe atoms diffuse to the surface, vacancies are accumulating inside the bare Fe clusters which leads to hollow structures eventually.^36^ However, no evidence of such cavities is found in our TEM studies. We therefore assume that the whole Fe@Au cluster undergoes restructuring during the oxidation process. Metallic nanoparticles tend to establish a spherical shape *via* self-diffusion along the particle surface in order to reduce the surface energy,^42^ but it is up to now uncertain why a relaxation seems to take place in the FeAu system, whereas it does not occur in pure Fe clusters where oxidation is typically described by the Kirkendall effect.^17^ Since the oxide layer itself acts as a barrier for further oxidation the growth process is self-limiting. The minimum thickness for a sufficient passivation of the iron core has been measured to be 3–5 nm of iron oxide.^15^

**Fig. 2:**
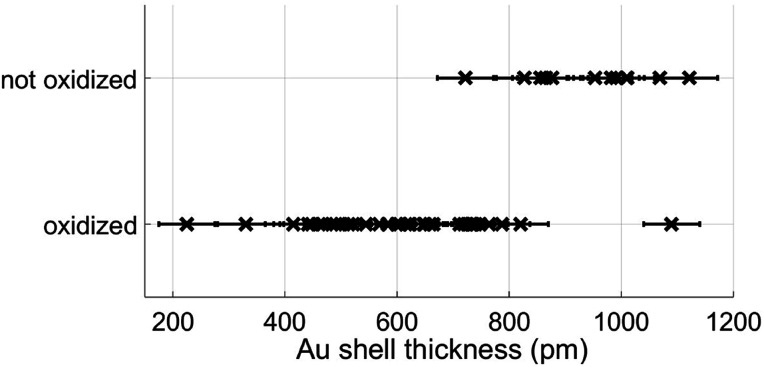
Classification results of nanoparticles as oxidized or not, plotted as a function of approximative minimum Au layer thickness.

**Fig. 3:**
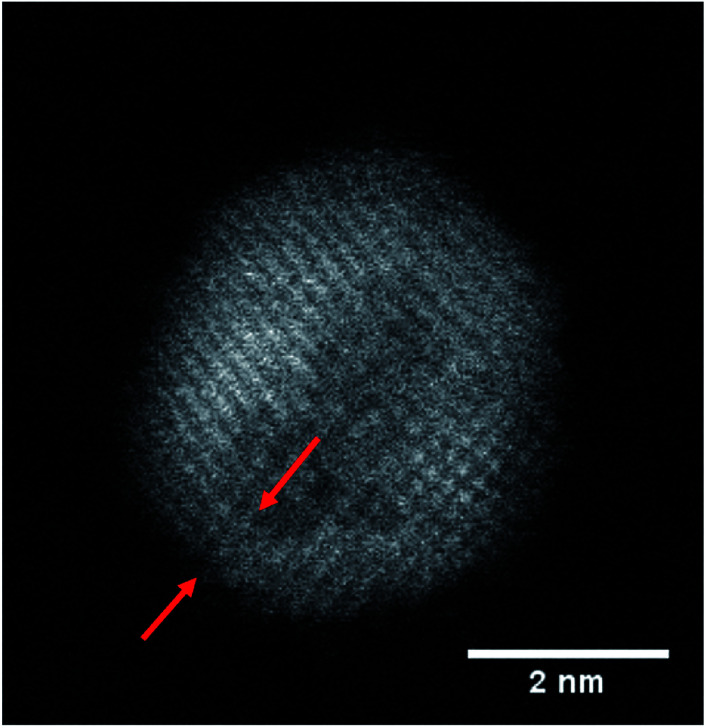
HAADF image of an Fe@Au cluster without signs of oxidation. The lattice structure of the Au-coating is clearly visible; in the lower left corner its thickness is approximately three atomic layers (marked by red arrows).

However, most relevant in this context is the ability to prevent any oxidation at all and keep the Fe core fully intact. Apparently, the amount of Au coating has a hindering effect on the oxidation process, but the effectiveness of this additional diffusion barrier depends on the thickness of the layer. Note that incomplete oxidation of Au-coated Fe leads to onion structures of the type Fe@Au@Fe_*x*_O_*y*_. This suggests that it is not the diffusion of molecular oxygen through the Au layer, but the diffusion of Fe through the Au layer which enables oxidation. We therefore perform a statistical analysis of the distributions of bimetallic particles with oxidized and intact Fe cores as a function of the Au layer thickness. 52 clusters are classified as either oxidized or not by structural inspection and the thickness of their corresponding Au shell which is measured at its thinnest position visible in the TEM projection. We note that it is likely to miss positions of minimum thickness due to the two-dimensionality which is intrinsic to a measurement in projection. Even a particle appearing as coated might have flaws which are not visible in the image. It is not possible to determine the shell thickness at the top or the bottom of a given cluster, resulting in a possible overestimation of the critical thickness. Nevertheless, every particle can be clearly characterized as having *n* layers of Au or less somewhere on its surface. As can be seen in Fig. 2, there is practically not a single not-oxidized particle to be found in cases with a minimum thickness of approximately 8 Å or below. From this finding we conclude that a thickness of three layers represents the minimum for an effective passivation of the Fe core.^47^ As an example, a cluster with a minimum thickness of three Au layers in projection is shown in Fig. 3, featuring a fully intact iron core.

**Fig. 4:**
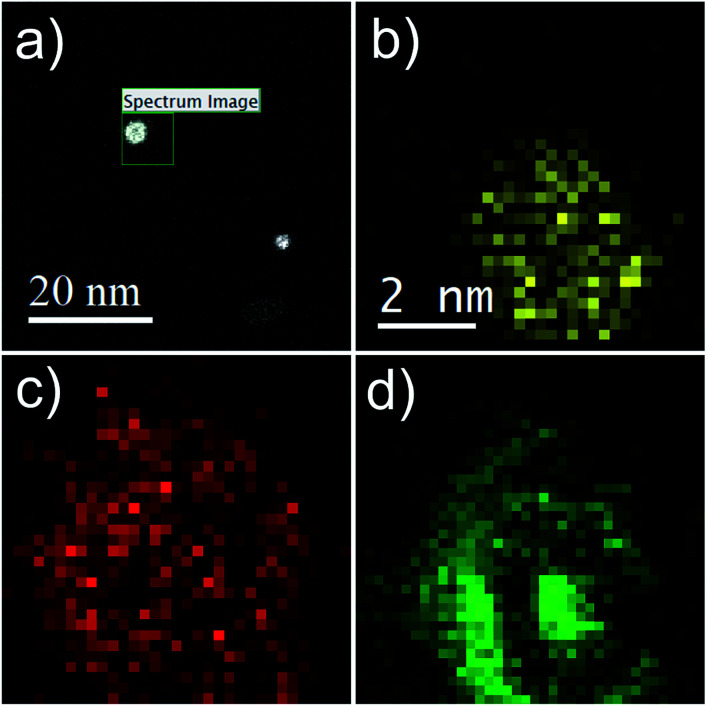
Spatially resolved EELS and EDX measurements of a three-layered system in the form of a core@shell@shell cluster. (a) Overview picture, showing some clusters after deposition, indicating the region of the elemental analysis. (b) EDX signal attributed to Au, (c) EELS map for O, and (d) the EELS map for Fe.

Additional proof of the multi-layered structure is provided by a series of EELS and EDX measurements. For the sake of a direct comparison, the same type of particle as shown in the HAADF picture (c) of Fig. 1 is chosen. Results are presented in Fig. 4. Note the reduced EELS intensity for O around the center of the particle, which clearly suggests an iron oxide distribution in the form of a spherical shell. This supports the assumption of a non-oxidized Fe core; otherwise, the oxygen signal would show a local maximum also somewhere near the center of the particle. The barely noticeable ‘halo’ in the HAADF image can be identified as a mixture of Fe and O, which also confirms the assumption of an iron oxide shell (see Fig. 1b–d).

### 3.1 Chemical analysis of the oxide

**Fig. 5:**
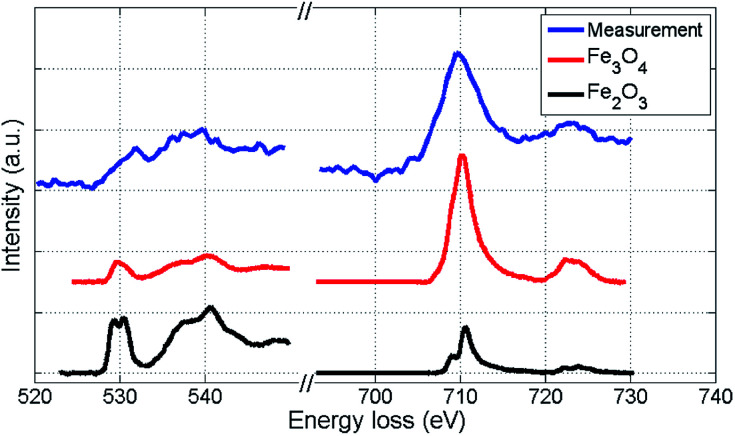
EELS spectra of the iron oxide shell of a Fe@Au@Fe_*x*_O_*y*_ cluster after background subtraction, compared to reference EELS spectra^48,49^ obtained for two different types of iron oxide. The O-K edge and the Fe-L_2,3_ edge are presented. Coinciding details of the Fe-L_2,3_ edge curvatures indicate that the largest part of the oxide-shell is Fe_3_O_4_.

We further attempt to identify the type of iron oxide present in the outmost layer after oxidation by EELS spectra analysis based on the O-K and Fe-L_2,3_ binding edges. Our spectra are presented in Fig. 5 together with two reference spectra obtained for two different types of iron oxide.^48,49^ By direct comparison of the curvature, the measured spectra (blue) show more similarity to the Fe_3_O_4_ profile than to the Fe_2_O_3_ profile. Slight deviations of the O-K edge spectra are due to the lower resolution in our measurements, which is a consequence of the low count number. Therefore, the actual characterisation of the iron oxide is rather based on the Fe-L_2,3_ edge (right spectra in Fig. 5), which allows an approximate characterization of the oxide. The EELS signal clearly indicates a distinct contribution of Fe_3_O_4_, but due to the possibility of overlapping signals in the relevant region, a mixed occurrence of both oxide structures can not be fully excluded.

We note that Wang *et al.* observed a similar EELS signal^50^ for the O-K edge of iron oxide clusters as measured in our experiment and assigned it to Fe_3_O_4_, confirming the findings of ref. 51. Studying the oxidation of small iron clusters, Peng *et al.* also found Fe_3_O_4_ as the prevailing product.^52^ Signorini *et al.* show that the composition rate of Fe_2_O_3_ and Fe_3_O_4_ depends on the size and structure of the original Fe cluster before oxidation.^53^Ref. 36 reports on a preeminence of Fe_2_O_3_, but notes a strong dependence on the oxidation conditions. Therefore, the choice of synthesis as well as oxidation method clearly affect the final outcome. Note that the structures presented here and in ref. 50 are studied *via* particle deposition, whereas ref. 36 employs a standard synthesis method in solution. Regarding a possible impact of the TEM electron beam on the oxidation process, we consider the amount of deposited energy as too small to cause such large changes in the structure. The total dosage was kept at a minimum to avoid structural effects due to electron impact.^54,55^

We further did not observe any effects of melting upon electron beam exposure. All images provided in the manuscript show a fully intact lattice structure, from which we conclude the presence of a solid phase. Additionally, our group published several *in situ* temperature transmission electron microscopy studies on comparable systems,^29,41^ where lattice structures remained clearly visible even at temperatures up to 400 °C.

### 3.2 Theoretical results

**Fig. 6:**
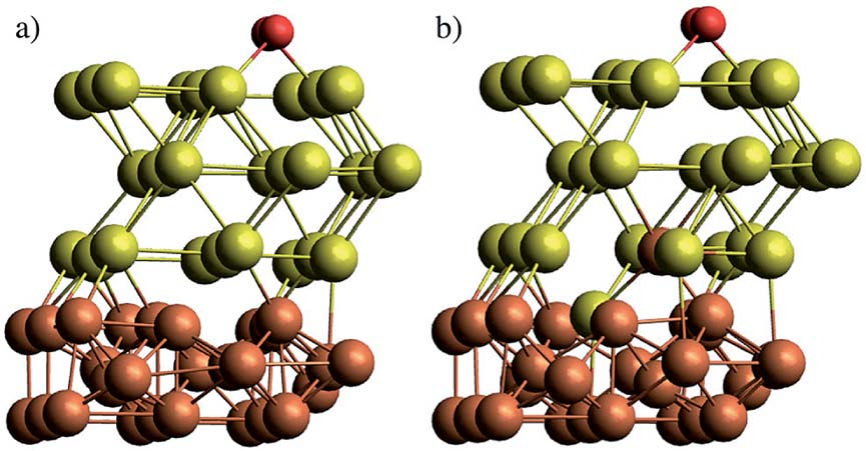
Slab models of Fe@Au core@shell clusters, comprising three layers of bcc Fe and three layers of fcc Au, with a single oxygen molecule adsorbed to the Au surface. (a) Pristine structure, (b) structure after swapping of a single Fe and a Au atom at the interface.

In a first attempt to study the passivating effect of Au on the Fe core *via* a computational approach we investigate the energy costs for the swapping of a single Fe atom with a Au atom near the interface of gold-coated bulk iron in the presence of molecular oxygen adsorbed to the bulk surface. This is done *via* periodic DFT calculations on a surface slab consisting of three atomic layers of iron and *n* = 1,2,3 layers of gold, treated with the PBE functional^56^ in combination with projected-augmented-wave (PAW) pseudopotentials^57,58^ as implemented in the Quantum Espresso suite of programs.^59^ We note that a similar approach had been undertaken by us in a previous study on the stability of NiAu clusters in the presence of oxygen,^29^ where we used at finite, icosahedral system consisting of a single Ni atom and 54 Au atoms to model smallest bimetallic clusters. In the current work, we aim for a more general discussion of the diffusion process with a better representation of the typical metallic interface of larger clusters. The supercell contains 9 metal atoms per layer. The box dimension in *x* and *y* is 8.598 Å, according to the experimental lattice constant of bcc iron. For the *z* or (001) direction, a length of 20 Å has been chosen to fully decouple the surface images, leaving approximately 10 Å of vacuum above after O_2_ adsorption on the surface. The gold layers can be interpreted as approximately fcc-structured, with the (001) Au plane interfacing the (001) plane of Fe at an angle of 45°. Energy cutoffs (130 Ryd for the energy, 1300 Ryd for the density) and *z*-distances have been tested in order to provide an accuracy of 0.05 eV or 1 kcal mol^−1^, which lies within the systematic error expected for the chosen functional. Energies are obtained *via* BFGS optimizations of the slab geometry after each interatomic swap. The lowest Fe layer is kept frozen to account for the bulk Fe structure. Converged slab structures consisting of 3 layers of Fe, three layers of Au and a single oxygen molecule attached to the surface are shown in Fig. 6. The left image shows the original structure, the right one the relaxed structure after a swapping of an Fe atom with an Au atom of lowest gold layer. All optimized DFT geometries are provided in the ESI† in Cartesian coordinate format.

**Fig. 7:**
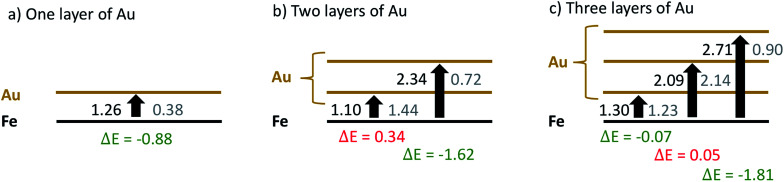
Comparison of energy costs for the swapping of a single Fe atom taken at the interface with Au atoms taken from various Au layers, calculated for gold coatings comprising (a) one, (b) two or (c) three Au layers, in units of eV. Values obtained without oxygen are printed in black, values obtained for geometries with a single oxygen molecule attached to the corresponding surface Au layer are printed in gray. For convenience, energy differences due to oxygen presence are printed below each arrow.

The energy differences obtained for the intermediate steps of Fe migration through the Au layers by interatomic swapping are presented in Fig. 7. In the following, we refer to the Au layer closest to the metallic interface as the first layer. The Fe atom involved in the commutation is always taken from the Fe layer closest to the interface. Energies are also compared between scenarios without (values printed in black) and with an O_2_ molecule physisorbed onto the gold surface (values printed in gray).

From the sequence of black values in each scenario it is clearly visible that a swapping is less feasible the further it takes place from the Fe–Au interface. Energy costs increase from 1.26 eV in the first to 2.71 eV in the third layer. However, swapping energies for a certain layer are practically independent of the total number of surface Au layers present in the model. For example, the energy costs for a swap in the first layer are only varying between 1.10 and 1.30 eV in all three models; a variation which we consider insignificant given the estimated accuracy of the functional used. The gray values printed in Fig. 7 reveal the impact of the oxygen molecule adsorbed to the upmost Au layer. In this case, a significant reduction of the Au/Fe swapping energy can be observed in all three models. This effect gets more pronounced with increasing layer index, producing energy differences of 0.88, 1.62 and 1.81 eV for the three models, respectively. It is also visible that a subsurface layer, if existent, is barely affected by the presence of O_2_: energies for a swapping in the first Au layer for the 2-layered system and in the second Au layer for the 3-layered system are only minimally affected. This finding is crucial for the overall interpretation of our experimental results: since swapping in the second Au layer is less feasible by more than 1 eV, this suggests that an intact subsurface layer of Au, *i.e.* a layer neither at the particle surface nor at the metal interface, is acting as the most effective barrier for Fe diffusion in the models under investigation. It can be realized only if at least three layers of Au are present, which agrees well with the experimental outcome.

We note that this simple, first approach towards the diffusion behavior between the metallic interface and the particle surface is only providing a qualitative interpretation for a series of reasons. First, it assumes a direct swapping of Au and Fe atoms at main lattice sites, which is energetically less feasible than *e.g.* diffusion along lattice defects. Second, it is restricted to this one type of diffusion, while several mechanisms and many alternative pathways though the lattice are possible. Third, and most importantly, our argument is built on intermediate state energies, while diffusion rates can only be related to transition states between these local minima. However, the aim of this theoretical investigation is to judge the impact of the O_2_ molecule on diffusion in general, and it can be expected that the presence of a subsurface layer of gold is critical as it represents a significantly high barrier for any type of Fe diffusion. Even if the actual mechanism of diffusion, most likely a complex interplay of lattice defects and possible diffusion pathways, is not yet revealed, it must involve intermediates as covered by this DFT study, and the trend of transition state energies must follow the trend of the calculated intermediate energies.

We note further that existing models such as the Cabrera–Mott theory^60^ have been successfully applied to the oxidation of bulk alloys^61^ as well as monometallic nanoparticles.^17^ Assuming charge separation effects in the oxide layer as a driving force for metal ion migration towards the surface, the Cabrera–Mott model is capable of predicting the oxidation sequence in alloys and provides estimates for oxidation rates.^61^ However, the model does not explicitly account for the structural consequences of these diffusion processes. In metallic nanoparticles, where the amount of oxidizable material is finite, the directed diffusion enforces the formation of cavities in the metal and leads to hollow, spherical metal oxide structures eventually.^17^ Interestingly, no such cavity formation can be observed in our experiment on Fe@Au core@shell structures. Obviously, the reason for this must lie in different lattice relaxation mechanisms for both scenarios, which points into the direction of computational simulations as the only way to understand structural effects of oxidation in metallic nanoparticles.

## 4 Conclusion

Fe@Au core@shell structures, synthesized *via* a sequential pickup of metal atoms by liquid helium nanodroplets, are deposited on TEM grids for HAADF imaging. After exposure to ambient air for 120 minutes, structural changes of the nanoparticles due to oxidation are studied. Various types of nanostructures are observed, including unaffected core–shell nanoparticles as well as clusters showing a partial or full oxidation of the Fe core, ranging from three-layered onion structures to Janus-particles. To the knowledge of the authors, this is the first time that Fe@Au@Fe-oxide clusters have been synthesized. Spatially resolved EDX and EELS measurements have been performed to confirm their chemical composition. A statistical analysis of the observed structures shows that a critical Au shell thickness of three atomic layers of Au seems a necessary condition for effective passivation of the sensitive Fe core. Density functional theory calculations on model systems comprising one, two or three protective Au layers on top of three layers of Fe indicate that an undisturbed monolayer of Au represents the largest barrier for Fe diffusion through the Au coating, while diffusion through the interface layer (directly above the Fe layer) or the surface layer (in the presence of molecular oxygen) is much less hindered. This explains the necessity for at least three Au layers, as it is observed in the experiment.

We hope that these findings will trigger future theoretical investigations *e.g. via* MD simulations with accurately parametrized force fields. Qualitative descriptions such as the Cabrera–Mott model, despite its predictive power with regards to oxidation rates and the sequence of oxides formed on a given alloy, do not provide information on the actual structural changes during oxidation at an atomic level. Yet, these structural changes are particularly important for a correct understanding of the reaction itself and its final outcome. While uncoated monometallic nanoparticles are known to produce hollow oxide structures when exposed to oxygen atmosphere, we do not see cavity formation in our experiments at all. From this we conclude that in the latter case, where the oxidation process is preceded by the diffusion of Fe through Au, a substantial reconfiguration of both metal phases is taking place simultaneously.

## Conflicts of interest

There are no conflicts to declare.

## Supplementary Material

NA-001-C9NA00161A-s001
