# Supplementary material for: On the passivation of iron particles at the nanoscale
Source: Nanoscale Adv. 2019 Apr 23;1(6):2276–83. doi: 10.1039/c9na00161a (PMC9418694; doi:10.1039/c9na00161a)
Supplement: NA-001-C9NA00161A-s001 [file NA-001-C9NA00161A-s001.pdf]

Electronic Supplementary Material (ESI) for Nanoscale Advances.
This journal is © The Royal Society of Chemistry 2019

Electronic Supplementary Information: On the passivation of iron particles on the nanoscale

Maximilian Lasserus,† Daniel Knez,† Martin Schnedlitz,† Andreas W. Hauser,*,† Ferdinand Hofer,† and Wolfgang E. Ernst*,†

†*Institute of Experimental Physics, Graz University of Technology, Petersgasse 16, A-8010 Graz, Austria. Fax: +43 (316) 873-108140; Tel: +43 (316) 873-8157; +43 (316) 873-8140;*
‡*Institute for Electron Microscopy and Nanoanalysis & Graz Centre for Electron Microscopy, Graz University of Technology, Steyrergasse 17, A-8010 Graz, Austria.*

E-mail: andreas.w.hauser@gmail.com; wolfgang.ernst@tugraz.at

In the first section of this Supporting Information we present a simplified graphical overview of our experimental setup. Section 2 contains a statistical evaluation of the species deposited on the TEM substrate. Section 3 discusses a model in which the core/shell ratio of ideal icosahedral clusters is analyzed, and uses this information to correlate doping rates to actual compositions for the various particle sizes. Section 4 lists the geometries of all intermediate states after atomic swapping as obtained from DFT optimizations.

Contents

1 Experimental setup **4**

2 Types of metallic particles and their distribution **5**

3 Closed shell model **6**

4 DFT geometries **7**

4.1 Geometries before O_2 adsorption 7

4.1.1 One layer 7

4.1.2 Two layers 8

4.1.3 Three layers 10

4.2 Geometries after O_2 adsorption 12

4.2.1 One layer 12

4.2.2 Two layers 14

4.2.3 Three layers 15

4.3 Swapped geometries, before O2 adsorption 17

4.3.1 One layer 17

4.3.2 Two layers, swap in 1st layer 19

4.3.3 Two layers, swap in 2nd layer 21

4.3.4 Three layers, swap in 1st layer 22

4.3.5 Three layers, swap in 2nd layer 24

4.3.6 Three layers, swap in 3rd layer 26

4.4 Swapped geometries, after O2 adsorption 28

4.4.1 One layer 28

4.4.2 Two layers, swap in 1st layer 30

4.4.3 Two layers, swap in 2nd layer 32

4.4.4 Three layers, swap in 1st layer 33

4.4.5 Three layers, swap in 2nd layer 35

4.4.6 Three layers, swap in 3rd layer 37

1 Experimental setup

A schematic of the He-droplet apparatus is presented in Figure 1. Our setup consists of three vacuum chambers, one for the helium droplet production (Source Chamber SC), one for the doping of the droplets (Pickup Chamber PC), containing the two evaporation cells filled with Fe and Au, and one for mass spectra analysis and the deposition on TEM grids (Main Chamber MC).

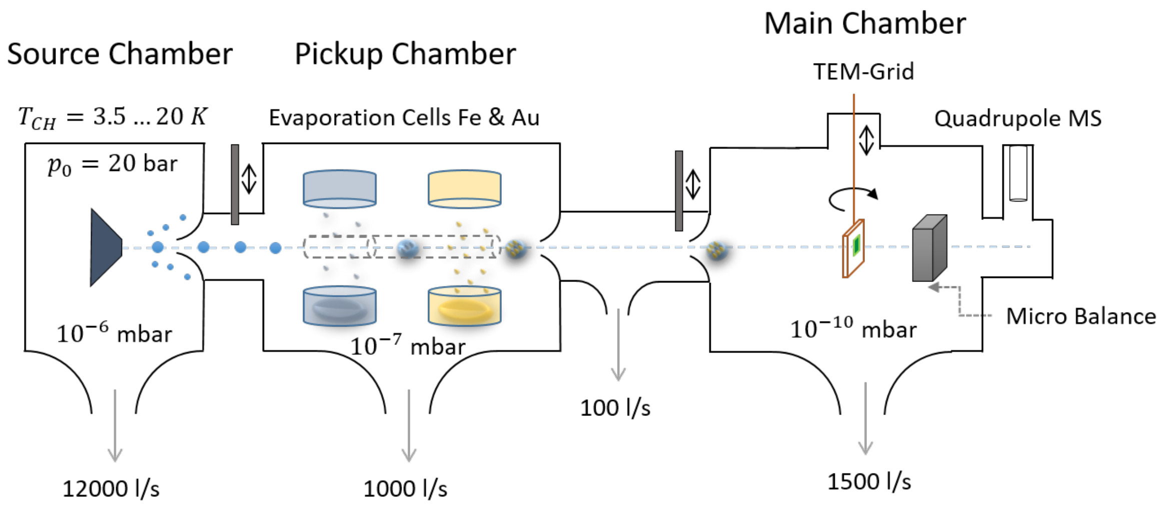

Figure 1: Schematic of the experimental setup used for the synthesis of Fe/Au nano structures inside liquid He droplets; see text for details.

2 Types of metallic particles and their distribution

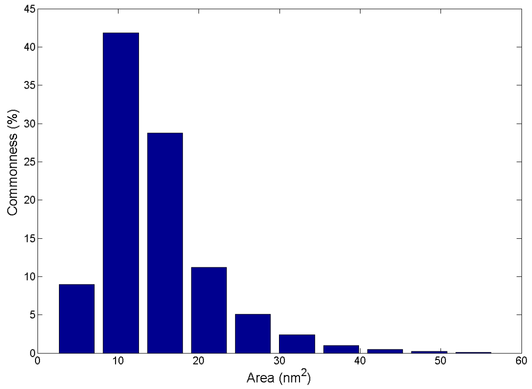

Figure 2: Histogram of sizes (area) of metallic particles synthesized and deposited in the current setup.

Figure 2 shows the size distribution of metallic structures deposited on the TEM grid in the course of He-mediated synthesis. The histogram is based on a classification of $\approx$ 1000 metal clusters (mixed and pure metal clusters). A pie chart of the types of mixed-metallic nanoparticles (i.e. excluding pure Fe ad Au clusters) is presented in Figure 3. It is based on a data set comprising $\approx$ 530 clusters whose structure could be clearly identified. In total, only 8 % of these mixed structures correspond to successfully passivated Fe cores, the rest is formed by the particularly interesting 3-layered, partially oxidized structures (32 %) and the fully oxidized particles (60 %). The latter can be further divided into core@shell and janus structures, showing a slight preference for the janus-type (36 %).

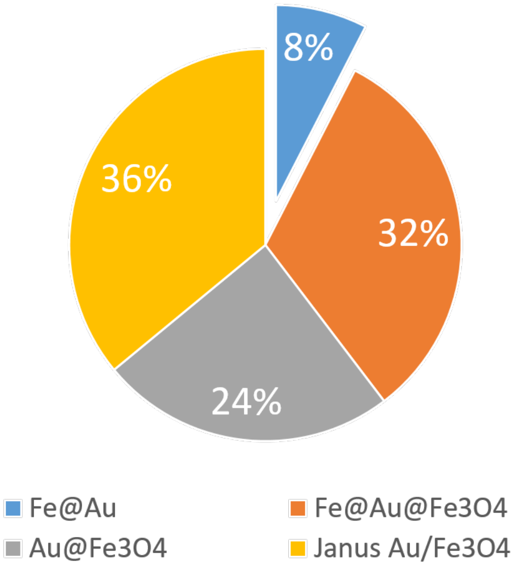

Figure 3: Distribution of nanostructures obtained via a two-step doping of helium droplets.

3 Closed shell model

The main manuscript mentions the variation of the doping rate as a convenient handle to control the distribution of Fe and Au the nano structures. Table 1 correlates the doping ratio with the number of of atoms in a cluster of certain size and its estimated number of Au layers. This estimation is based on a simple model assuming magic numbers of icosahedral core@shell geometries which are reasonably close to the desired ratio. It spans different cluster sizes from 1000 to 5000 atoms; the ratio is varied from 25 to 50 and 75 % Fe. Note that three additional shells as required for the effective passivation of the Fe core can be realized only in mixtures containing 25 % of Fe or less.

Table 1: Correlations between doping ratio, number of atoms, and estimated layers of shell material.

**clusters atoms**	**≈25% Fe**			**≈50% Fe**			**≈75% Fe**		
	N_{Fe}	N_{Au}	Sum	N_{Fe}	N_{Au}	Sum	N_{Fe}	N_{Au}	Sum
1000	147	776	923	561	854	1415	561	362	923
	3	*6*	*3 shells*	*5*	*7*	*2 shells*	*5*	*6*	*1 shell*
2000	561	1496	2057	923	1134	2057	1415	642	2057
	5	*8*	*3 shells*	*6*	*8*	*2 shells*	*7*	*8*	*1 shell*
3000	923	1946	2869	1415	1454	2869	2057	812	2869
	6	*9*	*3 shells*	*7*		*2 shells*	*8*	*9*	*1 shell*
4000	1415	2456	3871	2057	1814	3871	2869	1002	3871
	7	10	3 shells	8	10	2 shells	9	10	1 shell
5000	1415	3668	5083	2869	2214	5083	3871	1212	5083
	7	11	3 shells	9	11	2 shells	10	11	1 shell

4 DFT geometries

This section provides all bulk structures as obtained from partially constrained DFT optimizations with the PBE functional (Fe atoms are kept frozen); see main manuscript for details. We present all unperturbed geometries as well as all intermediate states, obtained by atomic swapping, i.e. exchanging a Fe atom from the interface layer with an Au atom of the upper layers. All geometries are given in Cartesian format in units of Ångstrom. Supercell dimensions x, y and z were set to 8.60 Å, 8.60 Å, and 20.00 Å, respectively. The formatting supports direct copy and paste from the pdf document.

4.1 Geometries before O_2 adsorption

4.1.1 One layer

```
36

Fe 0.000000000 0.000000000 0.000000000
Fe -0.209619780 0.101432951 2.361919896
Fe 0.000000000 2.866500000 0.000000000
Fe -0.105844327 2.974581928 2.277108325
Fe 0.000000000 5.733000000 0.000000000
Fe -0.030519771 5.545744462 2.314461000
Fe 2.866500000 0.000000000 0.000000000
Fe 3.073919673 -0.196329685 2.394530491
Fe 2.866500000 2.866500000 0.000000000
Fe 2.774344217 3.082720259 2.359460247
Fe 2.866500000 5.733000000 0.000000000
Fe 2.701097942 5.654822556 2.352494753
Fe 5.733000000 0.000000000 0.000000000
Fe 5.822752013 0.169838762 2.354752559
```

```
Fe 5.733000000 2.866500000 0.000000000
Fe 5.923519407 2.901441793 2.315029530
Fe 5.733000000 5.733000000 0.000000000
Fe 6.072987286 5.410251878 2.615357976
Fe 1.559467641 1.309371747 1.245748410
Fe 1.463620532 4.305106663 0.814899541
Fe 1.386210279 7.378023501 1.276193367
Fe 4.091789161 1.484223524 1.267997204
Fe 4.377445366 4.527618265 1.283609998
Fe 4.516330933 6.957180873 1.369631302
Fe 7.162960348 1.406445056 0.813213339
Fe 7.145597723 4.322040051 0.798472199
Fe 6.947610965 7.096762938 1.272334748
Au 4.295327840 1.468850823 4.045688588
Au 7.269660189 4.210437418 4.448993639
Au 4.418441012 7.061070770 4.086585108
Au 1.447826000 1.428956970 3.983614200
Au 4.316041976 4.262905222 4.132432196
Au 1.408986960 7.179907679 4.043121653
Au -1.418700529 1.450026172 4.218735341
Au 1.430264939 4.292346244 4.215005448
Au -1.383742936 7.162664516 4.136567735
```

4.1.2 Two layers

```
45

Fe 0.000000000 0.000000000 0.000000000
Fe -0.008512726 0.011923532 2.226893151
Fe 0.000000000 2.866500000 0.000000000
```

```
Fe -0.215166069 2.629546343 2.449489936
Fe 0.000000000 5.733000000 0.000000000
Fe 0.201282796 5.942237336 2.405106411
Fe 2.866500000 0.000000000 0.000000000
Fe 2.655054423 -0.182108666 2.404845094
Fe 2.866500000 2.866500000 0.000000000
Fe 3.121981954 3.000717603 2.402209267
Fe 2.866500000 5.733000000 0.000000000
Fe 2.821596870 5.782619803 2.240022913
Fe 5.733000000 0.000000000 0.000000000
Fe 5.984813032 0.205584128 2.449850667
Fe 5.733000000 2.866500000 0.000000000
Fe 5.726448332 2.874974640 2.230155562
Fe 5.733000000 5.733000000 0.000000000
Fe 5.580783813 5.483565252 2.404679104
Fe 1.524268249 1.630404007 1.270119516
Fe 1.304343029 4.140185880 1.273937151
Fe 1.425475446 7.178759460 0.809690630
Fe 4.228476171 1.226372352 1.271124105
Fe 4.251448129 4.342775343 0.838917480
Fe 4.456986948 7.303888986 1.276850936
Fe 7.141673125 1.456253485 0.817555241
Fe 7.362191974 4.391416670 1.271405359
Fe 6.982419079 7.061100658 1.270045970
Au 4.314729878 1.404266652 4.138873768
Au 7.193923135 4.292896665 4.139095640
Au 4.329005752 7.154011982 4.101295312
Au 1.454982221 1.428306998 4.136467246
Au 4.313290393 4.282503562 4.285939520
```

```
Au 1.449878399 7.154932682 4.296964930
Au -1.414016166 1.421157132 4.348125722
Au 1.444921088 4.274761335 4.101506572
Au -1.420022135 7.145137574 4.137231203
Au 0.038004390 2.881868958 6.270742327
Au 5.739043093 2.862835075 6.318863730
Au 2.860580677 2.831404568 6.257365796
Au 2.881732825 5.707482936 6.295753229
Au 8.600949408 5.704428343 6.263541202
Au 5.759733760 5.734722567 6.257410511
Au 0.034809852 8.576796172 6.323479643
Au 2.912092117 8.592327691 6.256880040
Au 5.735489924 8.557742730 6.272877239
```

4.1.3 Three layers

```
54

Fe 0.000000000 0.000000000 0.000000000
Fe 0.041880121 -0.046382325 2.279165911
Fe 0.000000000 2.866500000 0.000000000
Fe -0.297237884 2.575290914 2.449367195
Fe 0.000000000 5.733000000 0.000000000
Fe 0.073888899 5.941865461 2.345364950
Fe 2.866500000 0.000000000 0.000000000
Fe 2.870813752 -0.036902419 2.278509739
Fe 2.866500000 2.866500000 0.000000000
Fe 3.128788627 2.793216058 2.375510836
Fe 2.866500000 5.733000000 0.000000000
Fe 2.607545077 5.880792824 2.349030592
```

```
Fe 5.733000000 0.000000000 0.000000000
Fe 5.621268344 0.334827666 2.370754161
Fe 5.733000000 2.866500000 0.000000000
Fe 5.737633407 2.886870426 2.228768703
Fe 5.733000000 5.733000000 0.000000000
Fe 5.934491749 5.468962753 2.432429062
Fe 1.441282045 1.675446696 1.292861259
Fe 1.307154202 4.139425246 1.362105360
Fe 1.445446875 7.209594995 0.833862516
Fe 4.256536664 1.439477755 0.845952125
Fe 4.219018248 4.548119950 1.294301395
Fe 4.456370800 7.045033801 1.318565585
Fe 7.144467483 1.362532667 0.883108472
Fe 7.450793520 4.300372124 1.224004829
Fe 6.902898077 7.202659348 1.339688059
Au 4.272919886 1.440416697 4.293840282
Au 7.218971708 4.224940944 4.276617851
Au 4.311270405 7.161549657 4.110342657
Au 1.429977859 1.454086896 4.151980256
Au 4.312994922 4.320129195 4.198544139
Au 1.424694041 7.218717416 4.271736466
Au -1.479478089 1.354724558 4.322373059
Au 1.443323797 4.293349061 4.126362220
Au -1.460763535 7.143963504 4.167074173
Au 0.021947589 2.843273956 6.351035741
Au 5.726109397 2.852163196 6.385632039
Au 2.849457523 2.884747369 6.331285613
Au 2.884402771 5.737343678 6.309236526
Au 8.612894048 5.714534647 6.334772902
```

```
Au 5.745344076 5.720235062 6.319870281
Au -0.002705799 8.583977219 6.364397898
Au 2.864036529 8.604241077 6.347433295
Au 5.715573364 8.549715459 6.362997362
Au -1.437937138 1.400015941 8.406958656
Au 4.274762500 1.429312644 8.392987023
Au 1.426348947 1.442503365 8.429223851
Au -1.419051707 4.280435683 8.389521494
Au 4.299049301 4.311923097 8.382935310
Au 1.448113661 4.296640462 8.407083497
Au -1.429846915 7.131019467 8.411296278
Au 1.454067452 7.156531740 8.360690938
Au 4.312673226 7.158364202 8.419753735
```

4.2 Geometries after O_2 adsorption

4.2.1 One layer

```
38

Fe 0.000000000 0.000000000 0.000000000
Fe -0.109908134 -0.281251175 2.399569282
Fe 0.000000000 2.866500000 0.000000000
Fe 0.219368053 3.113815165 2.463819847
Fe 0.000000000 5.733000000 0.000000000
Fe -0.002036562 5.721454011 2.226057140
Fe 2.866500000 0.000000000 0.000000000
Fe 2.979787020 0.162902268 2.351755556
Fe 2.866500000 2.866500000 0.000000000
Fe 2.909520866 2.783019646 2.281065196
```

```
Fe 2.866500000 5.733000000 0.000000000
Fe 2.516737423 5.660967993 2.412876654
Fe 5.733000000 0.000000000 0.000000000
Fe 5.640298701 0.119482915 2.283623208
Fe 5.733000000 2.866500000 0.000000000
Fe 5.530003325 2.701884591 2.385887960
Fe 5.733000000 5.733000000 0.000000000
Fe 6.021679781 5.781802898 2.385308890
Fe 1.191724855 1.390462212 1.290611263
Fe 1.485960307 4.264257727 0.869620678
Fe 1.535295685 7.279487424 1.059874068
Fe 4.295851757 1.428805021 0.808657036
Fe 4.293209852 4.523355753 1.334512796
Fe 4.265067398 6.960857314 1.330559580
Fe 7.348795611 1.527336403 1.286748619
Fe 7.103192272 4.061050174 1.239586424
Fe 7.094421615 7.207734547 0.882494445
Au 4.281840929 1.422977709 4.261395319
Au 7.148435513 4.237972213 4.116634052
Au 4.290028617 7.150165675 4.008116215
Au 1.416837584 1.427000266 4.088435928
Au 4.276357855 4.313201184 4.029486331
Au 1.421528473 7.211520172 4.150079429
Au -1.407907265 1.420592060 4.039898921
Au 1.416332924 4.318235760 4.324970727
Au -1.442980318 7.158236247 4.231523764
O 4.240776928 5.091229150 6.105844727
O 4.276844338 6.414763895 6.100345150
```

4.2.2 Two layers

```
47

Fe 0.000000000 0.000000000 0.000000000
Fe -0.144168923 0.237304005 2.379623187
Fe 0.000000000 2.866500000 0.000000000
Fe -0.016075003 2.856405799 2.238855783
Fe 0.000000000 5.733000000 0.000000000
Fe 0.239136119 5.480101031 2.466886674
Fe 2.866500000 0.000000000 0.000000000
Fe 2.962881816 -0.240925681 2.385375175
Fe 2.866500000 2.866500000 0.000000000
Fe 2.620841180 3.122803218 2.470675653
Fe 2.866500000 5.733000000 0.000000000
Fe 2.864040464 5.727987604 2.233982594
Fe 5.733000000 0.000000000 0.000000000
Fe 5.781142966 0.041840752 2.249098173
Fe 5.733000000 2.866500000 0.000000000
Fe 5.973354664 2.736722726 2.388946206
Fe 5.733000000 5.733000000 0.000000000
Fe 5.482264425 5.851209996 2.393595551
Fe 1.653053002 1.394750864 1.266613641
Fe 1.433722953 4.304037062 0.823369158
Fe 1.207306042 7.217469481 1.278925930
Fe 4.148230162 1.569344855 1.298988923
Fe 4.356739406 4.080279615 1.266302758
Fe 4.332927739 7.205422386 0.847534313
Fe 7.140068709 1.408228943 0.825498181
```

```
Fe 7.103887820 4.519462336 1.272813942
Fe 7.300561918 7.013489056 1.291336449
Au 4.290168569 1.433903774 4.079412449
Au 7.162764174 4.311848081 4.164221230
Au 4.287317614 7.146682494 4.256998516
Au 1.442361015 1.429927642 4.141904613
Au 4.298156983 4.283841641 4.159479220
Au 1.412989404 7.158370556 4.159551511
Au -1.430793964 1.438614040 4.274789174
Au 1.429754906 4.312044826 4.389671253
Au -1.428679252 7.149537505 4.091127830
Au -0.026546481 2.854183814 6.324704778
Au 5.704813916 2.858640879 6.261486770
Au 2.880522924 2.808298268 6.275943706
Au 2.777669106 5.719874731 6.407754360
Au 8.564245311 5.752597845 6.377875855
Au 5.795095914 5.714654703 6.251485523
Au 0.010081105 8.579756536 6.242878258
Au 2.858053694 8.607187027 6.225243370
Au 5.734666615 8.602984482 6.250062146
O 4.237996541 5.951376432 8.145668387
O 4.245447643 5.060538658 9.063678655
```

4.2.3 Three layers

```
56

Fe 0.000000000 0.000000000 0.000000000
Fe 0.047649559 -0.061893452 2.285675345
Fe 0.000000000 2.866500000 0.000000000
```

```
Fe -0.301497639 2.574123197 2.457099527

Fe 0.000000000 5.733000000 0.000000000

Fe 0.076883351 5.940760070 2.353759375

Fe 2.866500000 0.000000000 0.000000000

Fe 2.899933691 -0.005838338 2.273688751

Fe 2.866500000 2.866500000 0.000000000

Fe 3.115007049 2.806133613 2.378230084

Fe 2.866500000 5.733000000 0.000000000

Fe 2.610159100 5.886494522 2.352715213

Fe 5.733000000 0.000000000 0.000000000

Fe 5.610251843 0.333389739 2.379466442

Fe 5.733000000 2.866500000 0.000000000

Fe 5.730104325 2.877191672 2.230912528

Fe 5.733000000 5.733000000 0.000000000

Fe 5.934405750 5.492991730 2.436059383

Fe 1.437547410 1.673537789 1.286335272

Fe 1.306893365 4.133436437 1.355723920

Fe 1.445102841 7.207703101 0.836127375

Fe 4.268288887 1.443641969 0.826209477

Fe 4.226554151 4.546585592 1.291027951

Fe 4.449487526 7.044485872 1.305460297

Fe 7.158789978 1.361754985 0.886544914

Fe 7.444661947 4.302175257 1.231693080

Fe 6.907084489 7.213641879 1.337072854

Au 4.280324120 1.463391147 4.291570474

Au 7.224214797 4.236417562 4.270536896

Au 4.314763930 7.178048679 4.109447027

Au 1.429993640 1.455635755 4.142221712

Au 4.327372847 4.334857952 4.189143767
```

```
Au 1.424480532 7.230876156 4.265191740
Au -1.480865771 1.370775910 4.337408479
Au 1.447526743 4.294912800 4.124326793
Au -1.457741918 7.163532444 4.167319624
Au 0.012517666 2.876358307 6.377242026
Au 5.726024135 2.892391794 6.418312437
Au 2.842867646 2.924466750 6.307924148
Au 2.871638041 5.780333298 6.320941416
Au 8.611612937 5.756191613 6.325876453
Au 5.729638136 5.758100316 6.309097111
Au -0.005475112 8.620141237 6.393654284
Au 2.855165218 8.648822705 6.330956363
Au 5.715259644 8.593786364 6.392696451
Au -1.417187922 1.443691195 8.418468693
Au 4.286768576 1.484883702 8.479323874
Au 1.446830278 1.504673675 8.502867032
Au -1.455596014 4.355309205 8.457671093
Au 4.320681084 4.267968873 8.393720862
Au 1.353460855 4.266905162 8.401769283
Au -1.439446183 7.153184439 8.453135466
Au 1.383100945 7.331073581 8.429589494
Au 4.338505029 7.319576354 8.431723403
O 3.553049047 5.777528280 9.929085556
O 2.164948477 5.811974077 9.932412828
```

4.3 Swapped geometries, before O2 adsorption

4.3.1 One layer

```
36
```

```
Fe 0.000000000 0.000000000 0.000000000
Fe 0.270910130 -0.246821697 2.563455821
Fe 0.000000000 2.866500000 0.000000000
Fe -0.006615561 3.118352458 2.392829997
Fe 0.000000000 5.733000000 0.000000000
Fe 0.075818877 5.703477137 2.309496868
Fe 2.866500000 0.000000000 0.000000000
Fe 2.814993411 -0.103319275 2.314162065
Fe 2.866500000 2.866500000 0.000000000
Au 2.894614367 2.820242326 2.661925042
Fe 2.866500000 5.733000000 0.000000000
Fe 2.721869146 5.654807487 2.514458192
Fe 5.733000000 0.000000000 0.000000000
Fe 5.541142411 0.132090795 2.389256485
Fe 5.733000000 2.866500000 0.000000000
Fe 5.596719339 2.715760581 2.356172519
Fe 5.733000000 5.733000000 0.000000000
Fe 5.972917935 5.728330369 2.415777400
Fe 1.181832189 1.400100324 1.253821610
Fe 1.454793968 4.302402228 0.930423999
Fe 1.432070080 7.171178472 0.864692673
Fe 4.268326451 1.400969857 0.879207140
Fe 4.358542100 4.440998844 1.206612987
Fe 4.327534391 6.913232445 1.320146295
Fe 7.331478482 1.291665683 1.300279394
Fe 7.047211555 4.218720936 1.023001149
Fe 7.182186066 7.429145777 1.261086549
Au 4.389455535 1.326942867 4.402890902
```

```
Au 7.110748280 4.278360634 4.208434040
Au 4.333239950 7.124187840 4.121160176
Au 1.408394856 1.298606040 4.323365633
Fe 4.509029490 4.561646672 3.594245758
Au 1.499203128 7.059894351 4.362411969
Au -1.379171885 1.415931122 4.037934963
Au 1.316758478 4.292191469 4.391402167
Au -1.408315081 7.181710875 4.169399063
```

4.3.2 Two layers, swap in 1st layer

```
45


Fe 0.000000000 0.000000000 0.000000000
Fe 0.007534236 0.003968563 2.235219101
Fe 0.000000000 2.866500000 0.000000000
Fe -0.057542771 2.611757793 2.406677447
Fe 0.000000000 5.733000000 0.000000000
Fe 0.202506261 6.008992442 2.453772272
Fe 2.866500000 0.000000000 0.000000000
Fe 2.613700187 -0.061095968 2.406898589
Fe 2.866500000 2.866500000 0.000000000
Au 2.966991779 2.966088989 2.615205681
Fe 2.866500000 5.733000000 0.000000000
Fe 2.881459932 5.764473267 2.327322814
Fe 5.733000000 0.000000000 0.000000000
Fe 6.014625524 0.193874954 2.455512227
Fe 5.733000000 2.866500000 0.000000000
Fe 5.757088652 2.889118965 2.330373160
Fe 5.733000000 5.733000000 0.000000000
```

```
Fe 5.417909188 5.415439144 2.518339264
Fe 1.466990047 1.470074786 0.940856430
Fe 1.191101109 4.322447045 1.246356308
Fe 1.468220250 7.166793243 0.865655369
Fe 4.325392475 1.191785814 1.248037601
Fe 4.301849137 4.305652802 0.845369280
Fe 4.450522022 7.293950026 1.314061701
Fe 7.158878525 1.473652241 0.871775239
Fe 7.295777454 4.449008582 1.314090844
Fe 6.993860740 6.991354878 1.314501725
Au 4.395329721 1.388050517 4.245625678
Au 7.183254298 4.328969318 4.091365935
Au 4.334680927 7.173147390 4.093942240
Au 1.406896556 1.404731499 4.301993216
Fe 4.403997941 4.396631208 4.188805073
Au 1.466561909 7.211215121 4.326117220
Au -1.385641061 1.458706103 4.325418580
Au 1.398710344 4.397018687 4.250985946
Au -1.403166571 7.189827529 4.174380012
Au 0.004418966 2.942445158 6.301734037
Au 5.699618483 2.990878737 6.183816563
Au 2.908184792 2.906300300 6.204627244
Au 3.003559779 5.700842363 6.191201250
Au 8.609944813 5.768375358 6.340503247
Au 5.784693213 5.771444602 6.212671107
Au 0.010807318 8.605769686 6.379338801
Au 2.942919604 8.600533670 6.308857176
Au 5.770336289 8.593368829 6.336202540
```

4.3.3 Two layers, swap in 2nd layer

```
45


Fe 0.000000000 0.000000000 0.000000000
Fe 0.164226852 0.177301545 2.365617540
Fe 0.000000000 2.866500000 0.000000000
Fe 0.081920088 2.595917792 2.421747841
Fe 0.000000000 5.733000000 0.000000000
Fe -0.239752984 5.973471243 2.352755016
Fe 2.866500000 0.000000000 0.000000000
Fe 2.766430511 0.057366352 2.340363785
Fe 2.866500000 2.866500000 0.000000000
Au 2.941438217 2.696668926 2.671594257
Fe 2.866500000 5.733000000 0.000000000
Fe 3.135920135 5.710985424 2.349697226
Fe 5.733000000 0.000000000 0.000000000
Fe 5.449646566 0.478753935 2.815521604
Fe 5.733000000 2.866500000 0.000000000
Fe 5.685140696 2.919716986 2.347248686
Fe 5.733000000 5.733000000 0.000000000
Fe 5.644999188 5.610019089 2.336372662
Fe 1.445959887 1.445119221 0.840226189
Fe 1.320073054 4.408928951 1.162576280
Fe 1.468226020 6.962482794 1.240726106
Fe 4.405840665 1.341140057 0.998629771
Fe 4.302027151 4.298249180 0.853305375
Fe 4.510456469 7.386474934 1.351810560
Fe 7.019312236 1.267950199 1.271771489
```

```
Fe 7.406173378 4.241955082 1.289842575
Fe 6.911218002 7.476077470 1.446195583
Au 4.284565492 1.530856373 4.929569295
Au 7.162430666 4.383047495 4.116490623
Au 4.246309943 7.241532442 4.213653099
Au 1.368375588 1.432794237 4.608294252
Au 4.351510361 4.430705132 4.317140323
Au 1.397447207 7.185068404 4.083869429
Au -1.458800358 1.555351647 4.492483458
Au 1.348694704 4.446453513 4.034050735
Au -1.527140799 7.296904773 4.147222655
Au 0.021911895 3.372245650 6.354372818
Au 5.777806135 3.393723314 6.526164611
Au 2.772749922 3.514238355 6.447250898
Fe 1.401066058 5.835401343 6.310719557
Au 7.286635559 5.797921827 6.770389886
Au 4.290568793 5.833833811 6.873171033
Au 0.006028108 8.219485046 6.412988752
Au 2.774677831 8.133704603 6.474551941
Au 5.754267804 8.252779643 6.466406702
```

4.3.4 Three layers, swap in 1st layer

```
54

Fe 0.000000000 0.000000000 0.000000000
Fe -0.016664012 -0.012936039 2.240889637
Fe 0.000000000 2.866500000 0.000000000
Fe -0.058430084 2.571785321 2.385205267
Fe 0.000000000 5.733000000 0.000000000
```

```
Fe 0.229452024 6.004031171 2.447522619
Fe 2.866500000 0.000000000 0.000000000
Fe 2.572741279 -0.055113275 2.383952575
Fe 2.866500000 2.866500000 0.000000000
Au 2.996221218 2.995568719 2.618400878
Fe 2.866500000 5.733000000 0.000000000
Fe 2.857257900 5.792481842 2.320236476
Fe 5.733000000 0.000000000 0.000000000
Fe 6.001535885 0.230834442 2.450905101
Fe 5.733000000 2.866500000 0.000000000
Fe 5.795050795 2.849199176 2.319562837
Fe 5.733000000 5.733000000 0.000000000
Fe 5.413648401 5.411851640 2.539906668
Fe 1.474328095 1.476011842 0.954351979
Fe 1.184624134 4.322654112 1.255505389
Fe 1.478216073 7.158598050 0.870858810
Fe 4.321643739 1.182998212 1.257059712
Fe 4.313805472 4.312110920 0.858868534
Fe 4.467645912 7.284100708 1.348612706
Fe 7.160321117 1.474311339 0.866669048
Fe 7.289456476 4.462129308 1.346859535
Fe 6.984406231 6.987039799 1.372523893
Au 4.401693524 1.405539803 4.293171303
Au 7.181534982 4.314698096 4.125496305
Au 4.317891166 7.179679259 4.127407471
Au 1.428476118 1.428537034 4.312620586
Fe 4.431239855 4.430259237 4.219912333
Au 1.456673914 7.226794732 4.355721059
Au -1.373908450 1.455098623 4.357926181
```

```
Au 1.405208256 4.406619977 4.294599467
Au -1.423601359 7.174156048 4.193861136
Au 0.041027508 2.926452530 6.385945377
Au 5.750201698 2.951259997 6.311430725
Au 2.925851925 2.927931366 6.289883847
Au 2.955313103 5.754176581 6.315497339
Au 8.608191962 5.767511850 6.384804373
Au 5.763039658 5.758142365 6.281670382
Au 0.025598355 8.628774163 6.446154293
Au 2.923005713 8.639508805 6.389225889
Au 5.762650364 8.603324493 6.382832297
Au -1.429459057 1.495646420 8.384015393
Au 4.316189603 1.492289212 8.403662251
Au 1.475323793 1.490400269 8.443677398
Au -1.392899885 4.337943471 8.428591241
Au 4.352023689 4.347240654 8.380179463
Au 1.495617676 4.331262703 8.401534791
Au -1.418059361 7.175493442 8.435507963
Au 1.501556804 7.186628902 8.391951309
Au 4.345211430 7.202064809 8.435956433
```

4.3.5 Three layers, swap in 2nd layer

```
54

Fe 0.000000000 0.000000000 0.000000000
Fe 0.017788561 -0.018919922 2.239379267
Fe 0.000000000 2.866500000 0.000000000
Fe -0.011300299 2.584072292 2.385512204
Fe 0.000000000 5.733000000 0.000000000
```

```
Fe 0.293418560 5.980669802 2.518733571
Fe 2.866500000 0.000000000 0.000000000
Fe 2.544604487 -0.042102259 2.426781223
Fe 2.866500000 2.866500000 0.000000000
Au 2.897013658 2.939437752 2.592377018
Fe 2.866500000 5.733000000 0.000000000
Fe 2.849131367 5.816470764 2.297069928
Fe 5.733000000 0.000000000 0.000000000
Fe 6.047087058 0.123973893 2.409234539
Fe 5.733000000 2.866500000 0.000000000
Fe 5.798854196 2.882986002 2.284005527
Fe 5.733000000 5.733000000 0.000000000
Fe 5.475541838 5.585614519 2.389846286
Fe 1.461696769 1.449306479 0.917560297
Fe 1.185380526 4.334091339 1.237719533
Fe 1.467353232 7.153825934 0.879352658
Fe 4.322913187 1.195031240 1.273066256
Fe 4.314744876 4.301525767 0.863839466
Fe 4.351900188 7.382691036 1.340675025
Fe 7.135577091 1.486361997 0.875812653
Fe 7.305035271 4.466908517 1.359671982
Fe 7.122200831 6.921677483 1.345978196
Au 4.400170933 1.355542087 4.249617229
Au 7.182309855 4.341698004 4.137834777
Au 4.326018415 7.170120377 4.194525830
Au 1.411084912 1.384062000 4.366095968
Au 4.303010240 4.381911785 4.436104136
Au 1.521512070 7.161023316 4.444970975
Au -1.388482235 1.463917204 4.305081877
```

```
Au 1.408356897 4.388864103 4.391536762
Au -1.385754551 7.178471567 4.230238726
Au 0.011385323 2.897546952 6.415132050
Au 5.782908322 2.881933259 6.389299535
Au 2.896005221 2.883845147 6.360424032
Fe 2.868870285 5.758073206 6.541628719
Au 8.622695790 5.764700002 6.412254091
Au 5.736517384 5.788550652 6.347961175
Au 0.022550431 8.630534361 6.466326040
Au 2.952117901 8.559553298 6.389737341
Au 5.792466661 8.619696527 6.399404496
Au -1.401073625 1.486405344 8.452253538
Au 4.322499478 1.451464660 8.443969491
Au 1.516510241 1.453895039 8.458306576
Au -1.425133793 4.357674894 8.449984403
Au 4.290913540 4.350630907 8.330312228
Au 1.485451658 4.330024458 8.390148338
Au -1.409669514 7.174995335 8.462919165
Au 1.518870969 7.148690302 8.398032229
Au 4.325974329 7.153940577 8.410104704
```

4.3.6 Three layers, swap in 3rd layer

```
54

Fe 0.000000000 0.000000000 0.000000000
Fe -0.014822356 0.025475329 2.251291483
Fe 0.000000000 2.866500000 0.000000000
Fe -0.033653906 2.500106693 2.466055619
Fe 0.000000000 5.733000000 0.000000000
```

```
Fe 0.137740861 6.025823281 2.403860087

Fe 2.866500000 0.000000000 0.000000000

Fe 2.601516707 -0.044948455 2.392919709

Fe 2.866500000 2.866500000 0.000000000

Au 2.915720243 2.790626459 2.630708227

Fe 2.866500000 5.733000000 0.000000000

Fe 2.822827279 5.824911881 2.284328652

Fe 5.733000000 0.000000000 0.000000000

Fe 5.971163860 0.301778241 2.524417019

Fe 5.733000000 2.866500000 0.000000000

Fe 5.801576962 2.846233738 2.288290142

Fe 5.733000000 5.733000000 0.000000000

Fe 5.622773997 5.475616100 2.346377060

Fe 1.452320489 1.468685076 0.920949839

Fe 1.213048669 4.293083586 1.281939799

Fe 1.465823574 7.147498586 0.863275088

Fe 4.339486323 1.184849888 1.216069988

Fe 4.270570781 4.310308528 0.886774245

Fe 4.462769532 7.297495638 1.339343913

Fe 7.147705007 1.477593976 0.886030301

Fe 7.403363258 4.323439711 1.339072316

Fe 6.932433403 7.137733789 1.337597964

Au 4.375865524 1.312441046 4.462109699

Au 7.180857570 4.312773517 4.140368847

Au 4.333473368 7.194312307 4.089487586

Au 1.390128214 1.352951936 4.479353797

Au 4.352745688 4.357617413 4.304265319

Au 1.440760742 7.240341859 4.323784142

Au -1.403861843 1.409044290 4.489180292
```

```
Au 1.357614926 4.407448256 4.203454043
Au -1.431631234 7.206677402 4.194385941
Au 0.012685303 3.036106743 6.362371925
Au 5.776467176 3.037822765 6.372805859
Au 2.879193351 2.988874637 6.292108891
Au 2.902122692 5.839992671 6.269631691
Au 8.585682677 5.867026777 6.320450428
Au 5.769308608 5.897537750 6.280629446
Au 0.034002290 8.729882153 6.926015831
Au 2.895775681 8.704312368 6.750368659
Au 5.783156394 8.725589210 6.813551911
Au -1.417591308 2.001336576 8.606666051
Au 4.295008536 2.020030038 8.567679549
Au 1.511354962 2.093264743 8.600914948
Au 0.050706240 4.443618917 8.952455202
Au 5.641372000 4.464272048 8.912753273
Fe 2.883357600 4.446220455 8.474087774
Au -1.423743989 6.877456209 8.575925603
Au 1.507224329 6.788066227 8.548468514
Au 4.298892380 6.879208090 8.532005983
```

4.4 Swapped geometries, after O2 adsorption

4.4.1 One layer

```
38


Fe 0.000000000 0.000000000 0.000000000
Fe 0.180072115 0.014303273 2.329401694
Fe 0.000000000 2.866500000 0.000000000
```

```
Fe 0.200357082 2.478333678 2.585237419
Fe 0.000000000 5.733000000 0.000000000
Fe 0.070554499 5.859389114 2.368470929
Fe 2.866500000 0.000000000 0.000000000
Fe 2.788138402 -0.033696513 2.278027377
Fe 2.866500000 2.866500000 0.000000000
Au 2.913454338 2.759519788 2.674883255
Fe 2.866500000 5.733000000 0.000000000
Fe 2.752238232 5.664886227 2.410688458
Fe 5.733000000 0.000000000 0.000000000
Fe 5.505237275 0.267996972 2.393497230
Fe 5.733000000 2.866500000 0.000000000
Fe 5.547344483 2.778008160 2.389245435
Fe 5.733000000 5.733000000 0.000000000
Fe 5.966749907 5.481783413 2.373945317
Fe 1.433418617 1.475579445 0.865218905
Fe 1.249457730 4.254142747 1.204132729
Fe 1.428296446 7.129982315 0.846780929
Fe 4.298380222 1.409484132 0.815193421
Fe 4.312586974 4.444009952 1.096520853
Fe 4.447244011 7.028281526 1.299034496
Fe 7.198870397 1.585406906 1.210091058
Fe 7.406359245 4.105149962 1.347094426
Fe 6.971623544 7.282593688 1.334178311
Au 4.371343423 1.326161611 4.471344821
Au 7.206225291 4.099363403 4.106424701
Au 4.330835385 7.150659787 4.013838862
Au 1.432801843 1.270284643 4.496140250
Fe 4.442843305 4.542002988 3.752856577
```

```
Au 1.437217760 7.136005274 4.200108499
Au -1.442417946 1.295505039 4.165922634
Au 1.321255734 4.342074242 4.240513054
Au -1.432535577 7.135747080 4.067391810
O 4.430002190 4.761503913 5.504418030
O 4.416820537 6.042980314 5.931659976
```

4.4.2 Two layers, swap in 1st layer

```
47

Fe 0.000000000 0.000000000 0.000000000
Fe -0.250436504 0.270268721 2.456339110
Fe 0.000000000 2.866500000 0.000000000
Fe -0.096051646 2.830052305 2.268756514
Fe 0.000000000 5.733000000 0.000000000
Fe 0.243725486 5.488582651 2.405922350
Fe 2.866500000 0.000000000 0.000000000
Fe 3.107921425 -0.080508555 2.382134171
Fe 2.866500000 2.866500000 0.000000000
Au 2.811643198 2.927787100 2.615618448
Fe 2.866500000 5.733000000 0.000000000
Fe 2.928795165 5.766765439 2.319808356
Fe 5.733000000 0.000000000 0.000000000
Fe 5.714036245 0.037180029 2.272691752
Fe 5.733000000 2.866500000 0.000000000
Fe 5.772518058 2.722053753 2.476980694
Fe 5.733000000 5.733000000 0.000000000
Fe 5.552391366 5.997419599 2.474771919
Fe 1.429609270 1.210750765 1.227208969
```

```
Fe 1.429547602 4.293453485 0.851879617
Fe 1.278241305 7.306010702 1.285949509
Fe 4.290556104 1.465628528 0.958632873
Fe 4.532532781 4.315991012 1.292444690
Fe 4.276296323 7.140133968 0.864921078
Fe 7.152125032 1.449385273 0.842176773
Fe 6.996143033 4.465468969 1.325516006
Fe 7.362509452 7.078477018 1.309563239
Au 4.334401974 1.385499282 4.300420132
Au 7.211859144 4.340079068 4.134559571
Au 4.215280972 7.138298717 4.309892106
Au 1.343847272 1.412481531 4.288355980
Fe 4.555012531 4.406434676 3.790137635
Au 1.389962104 7.212806510 4.120491493
Au -1.456696703 1.476031867 4.345461116
Au 1.495563124 4.433414379 4.409698883
Au -1.434508987 7.200934260 4.171917561
Au 0.025227237 2.934029560 6.337202563
Au 5.744494609 2.892794593 6.269664610
Au 2.960897666 2.937514676 6.221463732
Au 2.749356845 5.760250670 6.484890306
Au 8.506768145 5.801484762 6.374254319
Au 5.671497751 5.634818610 6.120530695
Au -0.027061482 8.598877935 6.369105394
Au 2.810393944 8.638279420 6.287023412
Au 5.743787964 8.559845982 6.343786103
O 4.193967544 6.326491757 8.146816139
O 4.177316976 5.706110224 9.256117359
```

4.4.3 Two layers, swap in 2nd layer

```
47

Fe 0.000000000 0.000000000 0.000000000
Fe -0.275582106 0.227257244 2.452178880
Fe 0.000000000 2.866500000 0.000000000
Fe -0.085793841 2.818160058 2.270821355
Fe 0.000000000 5.733000000 0.000000000
Fe 0.268151336 5.516606044 2.428317662
Fe 2.866500000 0.000000000 0.000000000
Fe 3.155567237 -0.103137679 2.424776065
Fe 2.866500000 2.866500000 0.000000000
Au 2.813463275 2.932148849 2.622341668
Fe 2.866500000 5.733000000 0.000000000
Fe 2.882924107 5.793355907 2.288089226
Fe 5.733000000 0.000000000 0.000000000
Fe 5.726386942 0.023075326 2.239694304
Fe 5.733000000 2.866500000 0.000000000
Fe 5.797185756 2.602634847 2.412247722
Fe 5.733000000 5.733000000 0.000000000
Fe 5.519918317 6.006759143 2.482076303
Fe 1.434718894 1.196772095 1.263425676
Fe 1.433982392 4.291909734 0.852794318
Fe 1.304266548 7.330074140 1.304835840
Fe 4.264889372 1.452149704 0.936566832
Fe 4.551265791 4.316994525 1.265932543
Fe 4.266250176 7.133142558 0.848566925
Fe 7.178875936 1.471613463 0.836216639
```

```
Fe 7.036923596 4.456550347 1.325458885
Fe 7.307272674 6.999481283 1.326731841
Au 4.313808189 1.365789019 4.341252225
Au 7.182850397 4.348673244 4.119997617
Au 4.258606282 7.156150892 4.400506880
Au 1.320569429 1.397577167 4.287776553
Au 4.382962425 4.381277729 4.320727377
Au 1.408967351 7.216200718 4.116632284
Au -1.489787611 1.472035918 4.366376012
Au 1.478308245 4.425317053 4.457780139
Au -1.446684089 7.203865991 4.186312568
Au -0.079897410 3.007626131 6.351489664
Au 5.690046768 2.923346935 6.369296848
Au 2.814616696 2.837063065 6.313799581
Fe 3.157641301 5.616361143 6.513128597
Au 8.650995911 5.923145048 6.284800893
Au 5.839065812 5.790508775 6.367247262
Au -0.102747258 8.702762236 6.434886304
Au 2.715560388 8.482256331 6.315110829
Au 5.679545269 8.616680036 6.452704410
O 3.133267368 5.625911765 8.143238075
O 3.525984998 5.543177591 9.333845033
```

4.4.4 Three layers, swap in 1st layer

```
56

Fe 0.000000000 0.000000000 0.000000000
Fe 0.017195106 -0.017330069 2.235142292
Fe 0.000000000 2.866500000 0.000000000
```

```
Fe -0.027695702 2.587469430 2.386212094
Fe 0.000000000 5.733000000 0.000000000
Fe 0.255109624 6.000901814 2.463055201
Fe 2.866500000 0.000000000 0.000000000
Fe 2.599724215 -0.018472837 2.390257434
Fe 2.866500000 2.866500000 0.000000000
Au 2.959018261 2.970280748 2.602959007
Fe 2.866500000 5.733000000 0.000000000
Fe 2.869877534 5.778979964 2.320277288
Fe 5.733000000 0.000000000 0.000000000
Fe 6.048393838 0.186913392 2.450965937
Fe 5.733000000 2.866500000 0.000000000
Fe 5.735813640 2.934018207 2.346859361
Fe 5.733000000 5.733000000 0.000000000
Fe 5.432502358 5.437780894 2.535269129
Fe 1.450929631 1.465084696 0.917164242
Fe 1.184946762 4.323505438 1.243380719
Fe 1.491373149 7.158653448 0.873701564
Fe 4.350644480 1.191848297 1.246353276
Fe 4.312639456 4.322158655 0.860501304
Fe 4.453940916 7.313790851 1.355172667
Fe 7.134160600 1.492057642 0.902633792
Fe 7.303296668 4.458964767 1.348139523
Fe 7.011662122 6.965880278 1.350034981
Au 4.438583531 1.406850236 4.254619264
Au 7.206155101 4.303531972 4.118714755
Au 4.326990977 7.172926176 4.131164537
Au 1.433509692 1.426134998 4.328389419
Fe 4.469468246 4.385607987 4.188729920
```

```
Au 1.470656661 7.230633336 4.354553204
Au -1.350498058 1.446842143 4.349143468
Au 1.423848197 4.408927150 4.305158938
Au -1.401056734 7.160358446 4.192063753
Au 0.074324585 2.911227182 6.417676722
Au 5.793254732 2.924331059 6.337862932
Au 2.975842795 2.915656663 6.260318839
Au 2.989649842 5.739367017 6.315982726
Au 8.650639412 5.754245558 6.395606154
Au 5.781610050 5.730612457 6.255149505
Au 0.072731378 8.619742689 6.478293850
Au 2.977268833 8.637774411 6.359429602
Au 5.816362526 8.597659582 6.392820439
Au -1.381342804 1.485145230 8.402917984
Au 4.373439660 1.451776420 8.486347321
Au 1.546265560 1.457514542 8.512681253
Au -1.352700289 4.347435853 8.485109620
Au 4.450709436 4.215519739 8.404336264
Au 1.485898806 4.209308479 8.420191547
Au -1.345125963 7.136503485 8.476170160
Au 1.502089905 7.281087210 8.446830028
Au 4.462618689 7.275915861 8.417095744
O 3.680426417 5.716905766 9.914222515
O 2.277612232 5.745044322 9.905728622
```

4.4.5 Three layers, swap in 2nd layer

```
56

Fe 0.000000000 0.000000000 0.000000000
```

```
Fe 0.038008684 -0.008872816 2.263327700

Fe 0.000000000 2.866500000 0.000000000

Fe 0.027076775 2.612828840 2.387676698

Fe 0.000000000 5.733000000 0.000000000

Fe 0.347467098 5.998132480 2.554996685

Fe 2.866500000 0.000000000 0.000000000

Fe 2.586771896 0.038734339 2.410435874

Fe 2.866500000 2.866500000 0.000000000

Au 2.887784822 2.930988802 2.585182525

Fe 2.866500000 5.733000000 0.000000000

Fe 2.845486220 5.826842054 2.288449333

Fe 5.733000000 0.000000000 0.000000000

Fe 6.059002228 -0.115062281 2.448326230

Fe 5.733000000 2.866500000 0.000000000

Fe 5.717835207 3.085179877 2.356910531

Fe 5.733000000 5.733000000 0.000000000

Fe 5.527127973 5.660476405 2.370179059

Fe 1.426043164 1.454469002 0.894217479

Fe 1.191644713 4.338026185 1.218859654

Fe 1.449109883 7.190436035 0.860395789

Fe 4.438908568 1.262066788 1.255328547

Fe 4.303504233 4.334630067 0.866867011

Fe 4.326125171 7.435739820 1.338783116

Fe 6.981062817 1.500108576 1.131099473

Fe 7.333849853 4.457941097 1.366571205

Fe 7.214839260 6.886191490 1.301431261

Au 4.421247409 1.365477068 4.239699167

Au 7.244426096 4.338802726 4.148405560

Au 4.343463097 7.190141573 4.208652020
```

```
Au 1.415589303 1.415665487 4.386190360
Au 4.340641180 4.414366652 4.414902617
Au 1.563977583 7.211046928 4.433491571
Au -1.391094222 1.451370159 4.255876276
Au 1.471695088 4.430451053 4.412010170
Au -1.318947148 7.163619111 4.311833574
Au 0.044319381 2.910031347 6.407739554
Au 5.805454305 2.888732904 6.362337869
Au 2.936070718 2.879713329 6.361852466
Fe 2.909374282 5.829672824 6.462150063
Au 8.673169591 5.772431313 6.452956074
Au 5.779577183 5.791471130 6.375888602
Au 0.053440061 8.643191296 6.497644965
Au 2.985723022 8.566290397 6.412652076
Au 5.822302285 8.627696778 6.424265803
Au -1.351499156 1.504614896 8.524137135
Au 4.444212117 1.343036186 8.476665429
Au 1.481254456 1.350468482 8.487861072
Au -1.381640035 4.322339643 8.472589355
Au 4.337589014 4.394211585 8.302337629
Au 1.542344832 4.394343861 8.370112361
Au -1.380973539 7.177131430 8.469244044
Au 1.543356297 7.151535340 8.500351506
Au 4.348660368 7.145819038 8.478729708
O 3.648435077 2.749136397 10.037728021
O 2.274856006 2.733111268 10.034982738
```

4.4.6 Three layers, swap in 3rd layer

```
56
```

```
Fe 0.000000000 0.000000000 0.000000000
Fe 0.016354463 0.007024135 2.234328622
Fe 0.000000000 2.866500000 0.000000000
Fe -0.048077286 2.562296913 2.413114136
Fe 0.000000000 5.733000000 0.000000000
Fe 0.255829115 6.040245129 2.482723142
Fe 2.866500000 0.000000000 0.000000000
Fe 2.559808968 -0.006124697 2.418210925
Fe 2.866500000 2.866500000 0.000000000
Au 2.887866524 2.853835336 2.599633400
Fe 2.866500000 5.733000000 0.000000000
Fe 2.846129659 5.783042349 2.278415060
Fe 5.733000000 0.000000000 0.000000000
Fe 6.034921967 0.165420598 2.422574169
Fe 5.733000000 2.866500000 0.000000000
Fe 5.806783977 2.850957102 2.288598640
Fe 5.733000000 5.733000000 0.000000000
Fe 5.470936119 5.557267413 2.371311940
Fe 1.439754900 1.451654901 0.905425343
Fe 1.171552359 4.318941222 1.295758373
Fe 1.486314365 7.157939952 0.875697967
Fe 4.328547883 1.187190432 1.257279586
Fe 4.289529723 4.291790967 0.860445998
Fe 4.368812035 7.366573832 1.325054928
Fe 7.135533374 1.496116520 0.880602534
Fe 7.301665008 4.465911451 1.346601357
Fe 7.082012187 6.956598530 1.326941706
Au 4.380400182 1.322202681 4.315822722
```

```
Au 7.164101371 4.340960188 4.109191523
Au 4.324872790 7.180543548 4.135655008
Au 1.388155026 1.395313302 4.435631365
Au 4.323925833 4.337082038 4.356929110
Au 1.476283208 7.252655054 4.406278234
Au -1.399940930 1.432650604 4.373680743
Au 1.374100732 4.407995431 4.274103228
Au -1.414925107 7.204319639 4.214301398
Au 0.006611298 2.969823742 6.337073160
Au 5.768112751 2.937927569 6.358109484
Au 2.907481195 2.951847231 6.398510236
Au 2.915427889 5.733958219 6.428689972
Au 8.578818803 5.816581152 6.316595935
Au 5.759687423 5.788599559 6.323798615
Au 0.025906284 8.678774090 6.875350280
Au 2.844406676 8.650608429 6.539654967
Au 5.813362085 8.630698035 6.530529075
Au -1.477048004 1.885822350 8.553188940
Au 4.286639176 1.584733977 8.458816484
Au 1.448964862 1.987128415 8.590115677
Au -0.175340827 4.377577271 8.840865088
Au 5.338104663 4.368859304 8.731226590
Fe 2.552553194 4.370570407 8.793544100
Au -1.503200671 6.855238484 8.539196713
Au 1.419903521 6.766225948 8.558774617
Au 4.270199851 7.148861423 8.457434688
O 4.349226150 4.400415672 10.731617680
O 3.056505607 4.430231073 10.411410671
```
